# High prevalence of fecal carriage of extended-spectrum beta-lactamase producing *Enterobacterales* among patients with urinary tract infections in rural Tanzania

**DOI:** 10.3389/fmicb.2024.1517182

**Published:** 2025-01-06

**Authors:** Magreth Erick Macha, Weihong Qi, Salome N. Seiffert, Anja Bösch, Philipp Kohler, Honorathy Msami Urassa, Sabine Haller, Erin West, Maja Weisser Rohacek, Baharak Babouee Flury

**Affiliations:** ^1^St. Francis University College of Health and Allied Sciences, Ifakara, Tanzania; ^2^Medical Research Center, Kantonsspital St. Gallen, St. Gallen, Switzerland; ^3^Graduate School for Cellular and Biomedical Sciences, University of Bern, Bern, Switzerland; ^4^Functional Genomics Center Zurich, University of Zürich/ETH Zürich, Zürich, Switzerland; ^5^Division of Human Microbiology, Centre for Laboratory Medicine, St. Gallen, Switzerland; ^6^Division of Infectious Diseases, Infection Prevention, and Travel Medicine, Kantonsspital St. Gallen, St. Gallen, Switzerland; ^7^St. Francis Regional Referral Hospital, Ifakara, Tanzania; ^8^Clinical Trials Unit, Kantonsspital St. Gallen, St. Gallen, Switzerland; ^9^Division of Infectious Diseases, University Hospital Basel, Basel, Switzerland; ^10^Swiss Tropical and Public Health Institute, Allschwil, Switzerland; ^11^Ifakara Health Institute, Ifakara, Tanzania; ^12^Department of Infectious Diseases, Inselspital, Bern University Hospital, University of Bern, Bern, Switzerland

**Keywords:** fecal carriage, extended-spectrum beta-lactamase, enterobacterales, urinary tract infections, rural Tanzania

## Abstract

**Introduction:**

The global rise of extended-spectrum beta-lactamase-producing *Enterobacterales* (ESBL-PE) challenges resource-limited countries with insufficient laboratory infrastructure. This study investigates fecal carriage and risk factors for ESBL-PE and carbapenemase-producing organisms among patients with urinary tract infection (UTI) in rural Tanzania.

**Methods:**

This cross-sectional study was conducted at St. Francis Regional Referral Hospital, Ifakara, Tanzania, from October 2021 to August 2023, involving 326 UTI patients. Demographic data and resistance risk factors were collected via structured questionnaires. Stool samples collected pre-antibiotic treatment were screened for ESBL-PE and carbapenemase locally. Positive samples underwent further analysis in Switzerland using MALDI-ToF, Vitek MS, and whole-genome sequencing. Multivariable analysis assessed predictors associated with ESBL-PE carriage for risk factors with *p* < 0.05.

**Results:**

We enrolled 326 UTI patients (median age: 35.5 years, range: 25–52) and 189 (58.0%) were females. Fecal ESBL-PE colonization was detected in 70.9% of patients, predominantly *E. coli* (62.8%) and *K. pneumoniae* (33.0%). Whole-genome sequencing identified diverse phylogroups and sequence types, with CTX-M-15 being the most common ESBL gene. IncF plasmids were the primary carriers. Younger age (aOR: 0.98, 95% CI: 0.97–0.99; *p* = 0.0239) and inpatient status (aOR: 1.77, 95% CI: 1.08–2.91; *p* = 0.0036) were significant risk factors for ESBL-PE carriage.

**Conclusion:**

The high prevalence of ESBL-PE fecal carriage in rural Tanzania highlights the need for improved infection control and further research into community transmission dynamics.

## Background

Antimicrobial resistance (AMR) is a major global health challenge with significant consequences for public health and healthcare systems. In 2024, the Global Research on Antimicrobial Resistance (GRAM) project estimated that by 2050, there will be 1.91 million annual deaths globally attributable to AMR, with 6.63 million deaths attributable to AMR in sub-Saharan Africa. Without effective prevention measures, AMR will lead to a cumulative total of 39·1 million deaths attributable to AMR from 2025 to 2050 (Global Burden of Disease 2021 Antimicrobial Resistance Collaborators, 2024).

Reports indicate that the widespread fecal carriage of Extended-spectrum beta-lactamase producing *Enterobacterales* (ESBL-PE) is an emerging public health concern, contributing to infections in both community and hospital settings. Even in the absence of active infection, colonization with ESBL-PE remains a significant cause for concern (Godonou et al., 2022).

The high prevalence of fecal carriage of resistant pathogens facilitates the global transmission of resistance genes through travel, trade, and migration (Bokhary et al., 2021; Zamudio et al., 2024). In resource-limited settings with inadequate sanitation and hygiene infrastructure, the spread of resistance genes is exacerbated through contaminated food, water, and direct contact. This one health dynamic extends to animal populations via zoonotic spillover, cycling resistance genes back to humans through the environment or food chain (Aslam et al., 2021; Gebreyes et al., 2014). These mechanisms not only challenge effective treatment options but also contribute to increased morbidity and mortality due to limited therapeutic options (Iskandar et al., 2021).

The fecal carriage of ESBL-PE varies geographically, with prevalence ranging from 6 to 20% in Europe and North America, approximately 20% in the Eastern Mediterranean and Africa, and 24.5–27% in the Western Pacific and Southeast Asia in community and hospital settings (Bezabih et al., 2021; Pilmis et al., 2018; Islam et al., 2017).

In Africa, studies conducted in Chad and Rwanda have demonstrated a higher prevalence of ESBL-PE carriage in hospital settings than in the community (Ouchar Mahamat et al., 2019; Kurz et al., 2017). This pattern may be attributed to antibiotic use, antibiotic-induced gut dysbiosis, and reduced colonization resistance, which might promote ESBL-PE transmission through various routes, including person-to-person, contaminated food and water, and environmental exposure (Mai and Espinoza, 2023; Djuikoue et al., 2016).

In Eastern Africa specifically, ESBL-PE prevalence ranges from 6 to 17% in community settings and 38–83% in hospital environments (Storberg, 2014). A study conducted in East African hospitals has documented an overall pooled proportion of ESBL-PE at 42% with variation among countries: 61.7% in Uganda, 45.8% in Kenya, 38.8% in Tanzania and 30.9% in Ethiopia (Sonda et al., 2016).

Despite the growing concern about ESBL-PE in Tanzania, there is a notable lack of comprehensive studies on carriage of ESBL-PE across diverse age groups and clinical presentations (Silago et al., 2022; G et al., 2017; Ngowi et al., 2021; Mlugu et al., 2023). The available studies primarily focus on specific subpopulations or environments, leaving a fragmented understanding of the broader distribution of ESBL-PE. For instance, a study conducted among pregnant women reported a high prevalence of ESBL carriage rate of 64.3% (Mwandigha et al., 2020). Another study in urban informal settlements found that over 24% of ESBL-producing *E. coli* were isolated from private and shared latrines, indicating these facilities act as reservoirs for ESBL-PE transmission (Erb et al., 2018). Similarly, studies targeting hotel employees (Büdel et al., 2019) and healthcare settings (Manyahi et al., 2020) have provided valuable insights but are limited in scope. Most of these studies are urban-centric focus leaving rural communities underrepresented resulting in an incomplete picture of the epidemiology and dissemination of ESBL-PE in Tanzania. To address this knowledge gap, our study aims to evaluate the fecal carriage rate of and risk factors for multidrug-resistant *Enterobacterales* in patients presenting with symptoms of urinary tract infection at St. Francis Regional Referral Hospital (SFRRH) in Ifakara, a rural area in Tanzania. This research will contribute to the understanding of ESBL-PE epidemiology in the region and inform strategies for antimicrobial stewardship and infection control.

## Methodology

### Study design and study setting

**Figure 1:**
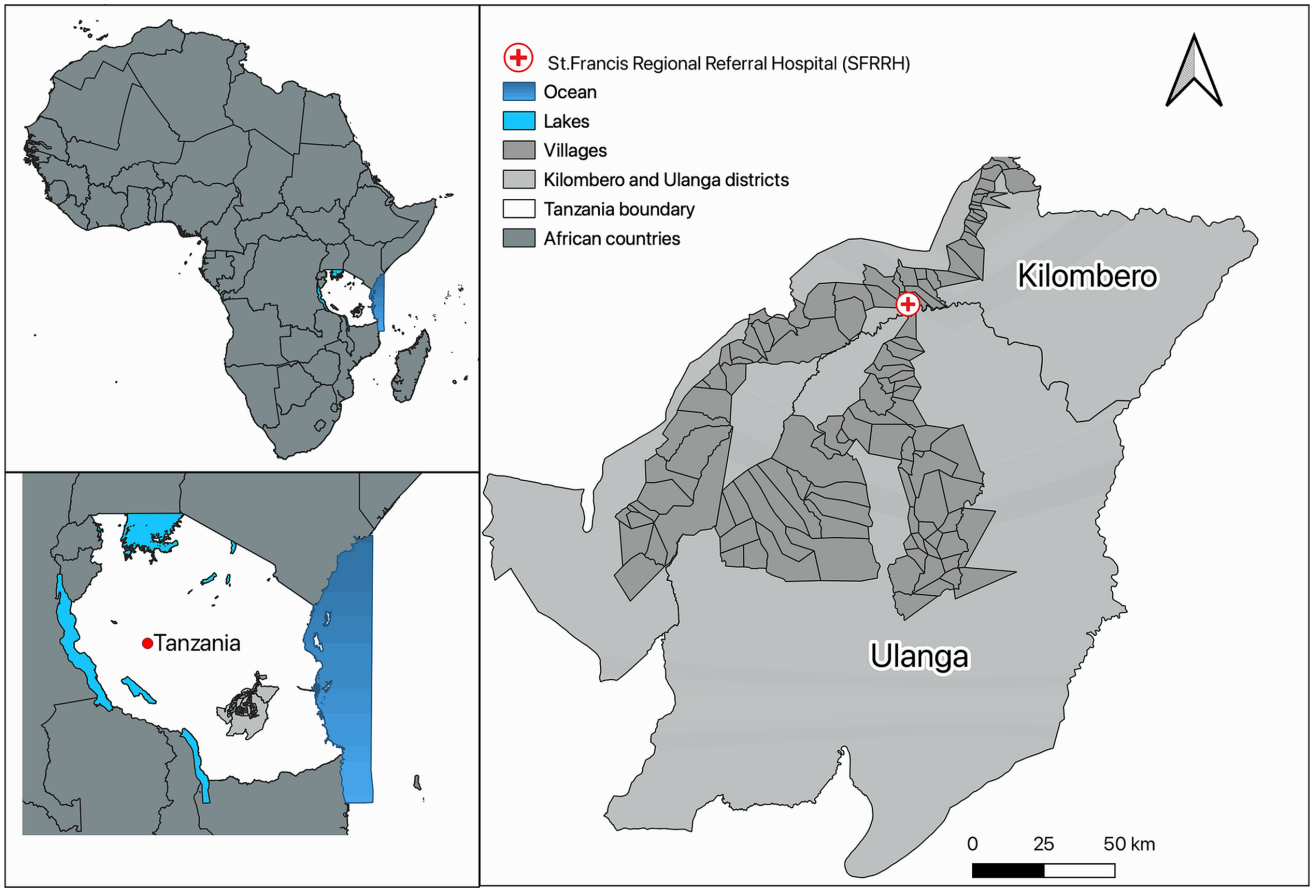
Map showing the location of St. Francis Regional Referral Hospital, Ifakara-Tanzania.

The study was conducted at St. Francis Regional Referral Hospital (SFRRH) in Ifakara, Tanzania, from October 2021 to August 2023. SFRRH is a 371-bed hospital with approximately 16,480 in-patient admissions, and 100,000 out-patient visits yearly serving a population of close to 1 million people in the Kilombero Valley. It is the main teaching hospital in South-Central Tanzania, playing a key role in promoting health and training human resources in that area (Figure 1).

The map was created using Quantum GIS v3.26 open source (Congedo, 2021).

### Patient recruitment

The study included in- and outpatients aged 18 years or older who presented with clinical evidence of urinary tract infection (UTI) according to the Infectious Diseases Society of America (IDSA) guidelines (Nicolle et al., 2019). All participants provided written informed consent. Ethical approval was granted by the National Health Research Committee of the Tanzania National Institute for Medical Research (certificate number NIMR/HQ/R.8a/Vol. IX/3759). Consent was obtained in Kiswahili. For illiterate individuals, clinicians or registered nurses provided detailed study explanations in the presence of a chosen witness, and willing participants indicated their consent with a thumbprint.

### Sample collection

Patients were instructed by a clinician or registered nurse to collect stool (Stool container, Mshale, Dar-es-Salaam, Tanzania). The samples were taken to the Microbiology laboratory at SFRRH within 2 h.

### Data collection and management

Structured questionnaires gathered socio-demographic and clinical data. Variables included age, gender, residence, history of past UTI, pregnancy status, history of diabetes, antibiotic use in the past 2 months, and companion animals. Data management and analysis plans were developed at SFRRH in Tanzania and Kantonsspital St. Gallen (KSSG) in Switzerland, respectively.

### Laboratory procedures

#### In Tanzania

Mid-stream urine of all patients who fulfilled the definition of UTI were collected aseptically and streaked on Blood, Cystine Lactose Electrolyte Deficient agar (CLED), and/or MacConkey agar [Liofilchem, Roseto degli Abruzzi (Te), Italy] using a calibrated 0.01 mL inoculating loop. Cultures underwent incubation for 18–24 h at 37°C. Agar plates exhibiting pure growth equivalent to or exceeding 10^5^ colony-forming units (CFU/mL) were categorized as having significant growth. Plates with no visible colonies were reported as having no bacterial growth. Results showing bacterial growth ≥10^2^ CFU/mL but below the threshold for significant bacterial growth of 10^5^ CFU/mL were considered insignificant. Cultures displaying more than two types of organisms were classified as mixed growth. The pure bacterial isolates underwent further identification based on colonial characteristics, microscopic features following Gram’s stain, and biochemical tests.

Stool samples underwent screening for extended-spectrum cephalosporin and carbapenem-resistant *Enterobacterales*. Briefly, the stool samples were enriched overnight, incubated for 18 h at 37°C, in Mueller Hinton broth containing cefuroxime (30 μg), and subsequently plated on CHROMagar™ ESBL and CHROMagar™ mSuperCARBA (CHROMagar, Paris-France). All colonies exhibiting variations in color and/or morphology were carefully selected and further inoculated on nutrient agar slants. These inoculated slants were then incubated for 18 h at 37°C and subsequently preserved in trypticase soy broth (TSB) containing 20% glycerol at −80°C.

For retrieval, bacteria were scraped from frozen bacterial glycerol stocks using a sterile loop. Subsequently, they were streaked onto nutrient agar plates and incubated for 24 h at 37°C. Following this incubation period, bacterial colonies were picked and inserted into a swab within the Stuart transport system (Trust lab, China). Tubes were labeled with specimen source and packed for shipment following the International Air Transport Association (IATA) regulations. A shipment was made after obtaining the Material Transfer Agreements (MTAs) and other shipping permits from the responsible authorities and were then shipped at room temperature to the Laboratory of Experimental Infectious Diseases at Kantonsspital St. Gallen (KSSG) in Switzerland for further analysis. Upon arrival at the laboratory, biosafety procedures for BSL-2 pathogens were followed. The isolates were processed in a Class II biosafety cabinet, workspaces and tools were disinfected before and after processing, and personal protective equipment gloves and lab coats were worn during handling. The isolates were kept frozen at −80°C for further use.

#### In Switzerland

##### Bacterial storage, identification, and susceptibility testing

The isolates were streaked on Luria Bertani agar (Sigma Aldrich, USA) and incubated at 37°C for 24 h. The following day, individual colonies were selected and then suspended in separate 1.5 mL tubes containing 1 mL of Luria Broth (Sigma Aldrich, USA) with 10% glycerol and stored in a − 80°C freezer for future experiments. The bacteria were retrieved for identification by using a sterile loop to scrape them off from a frozen bacterial glycerol stock.

The identification of isolates from urine was performed at the species level using MALDI-ToF mass spectrometry (MALDI Biotyper Smart System, Bruker Daltonics, Bremen, Germany). Based on the susceptibility test patterns obtained from the BD Phoenix™ M50 system (Becton Dickinson, Sparks, MD, USA), additional confirmation tests were performed *E*-test ESBL confirmation with specific *E*-test stripes, Carba-NP carbapenemase detection kit, all purchased from BioMérieux, Marcy l’Etoile, France and Carba-5 carbapenemase detection kit (NG Biotech, France).

For identification and antimicrobial susceptibility testing of isolates from stool, the VITEK^®^ MS PRIME MALDI-TOF (Matrix Assisted Laser Desorption Ionization-Time of Flight) system was employed. Additionally, the VITEK MS VITEK^®^ 2 COMPACT instrument, along with the VITEK^®^ 2 PC software and ready-to-use bacteria identification (ID) cards, as well as antibiotic susceptibility testing (AST) VITEK^®^ 2 ID/AST cards (N283, N240, P655), were utilized for the identification and antimicrobial susceptibility testing of the bacterial isolates (BioMérieux, Marcy L’ É toile, France). Additional susceptibility testing to confirm the presence of ESBL resistance mechanisms was conducted using the disk diffusion method in accordance with and interpreted according to European Committee on Antimicrobial Susceptibility Testing (EUCAST) guidelines (version 13.1, valid from 2023-06-29). Confirmation of carbapenemase producers was performed on a GeneXpert^®^ System (Cepheid, CA 94089, USA), utilizing the Xpert^®^ Carba-R test.

#### Nucleic acid extraction

Genomic DNA (gDNA) was extracted from all ESBL and carbapenemase-producing bacterial isolates derived from single colonies on plates. The QiaAMP Mini Kit (QIAGEN, Hilden, Germany) was employed for DNA extraction, following the manufacturer’s instructions, and the concentration was determined using the Nanodrop OneC spectrophotometer (Thermo Fisher Scientific, Massachusetts, United States). The extracted DNA was eluted in 50–100 μL of sterile water, and the DNA templates were stored at −20°C until further analysis.

### Whole genome sequencing

#### Library preparation

A total 1 ng of DNA from each sample was tagmented using Illumina Nextera XT according to standard protocol. Nextera adapters containing Unique Dual Indices (UDI) were added by PCR. The libraries were double-sided size selected and the quality and quantity of the libraries were validated using the Fragment Analyzer (Agilent, Santa Clara, California, USA). The libraries were normalized to 10 nM in Tris-Cl 10 mM, pH8.5 with 0.1% Tween 20 and pooled equimolar.

#### Cluster generation and sequencing

After library quantification, libraries were prepared for loading according to the NovaSeq workflow with the NovaSeq6000 Reagent Kit (Illumina, Catalog no. 20012865). Cluster generation and sequencing were performed on a NovaSeq6000 System with a run configuration of paired-end at 2 × 150 bp.

Illumina paired-end (PE) reads underwent quality checks using FastQC (v0.11.9) (Chen et al., 2018; Wingett and Andrews, 2018). Adapter sequences and low-quality read ends (identified with a sliding window of 4 bp and a base quality lower than Q20) were trimmed away using Fastp (v0.20.0). Trimmed reads were quality (Q20) and length (18 bp) were filtered using the same tool. Trimmed and filtered reads were mapped to the reference genome (Ensembl *Escherichia*_*coli* K12 MG1655 ASM584v2) using bowtie2 (v2.4.2). Variants were identified using samtools (v1.11)/bcftools (v1.11) (Li, 2012). A hierarchical cluster dendrogram based on pairwise identity-by-state (IBS) values from SNP data for all samples was computed using SNPRelate (v1.30.1) (Zheng et al., 2012). A phylogeny tree from SNP data was constructed using mashtree (v1.4.6) and combined with metadata using itol (v6).

Trimmed and filtered reads were assembled using Spades (v3.15.5) (Bankevich et al., 2012). Assembled contig sequences were annotated using prodigal (v2.6.1) (Hyatt et al., 2010). Annotated protein sequences were compared to the Swiss-Prot (downloaded on 2023/02/10) (Bairoch and Apweiler, 2000; Altschul et al., 1990). Phylogroups of assembled genomes were determined using the Clermont Typing pipeline (v20.03) and Ridom SeqSphere (v10.0.2) (Jünemann et al., 2013).

*E. coli* Achtman MLST database was downloaded from pubmlst (Roer et al., 2017). Annotated gene sequences were compared to the MLST database (downloaded on 2023/03/15 and 2023/08/30) using blastn (ncbi-blast v2.12.0+). A customized perl script was used to identify the MLST profile, associated ST, and clonal complex. In silico serotyping was performed using SerotypeFinder (v2.0.1). Fim type was determined using FimTyper (v1.1) (Jolley et al., 2018) by comparing assembled contig sequences against the FimTyper database using blast (2.12.0+). Antibiotic-resistant genes were predicted using fARGene (Fragmented Antibiotic Resistance Gene iENntifiEr) (v0.1) (Berglund et al., 2019) and blastp (2.12.0+) comparison against the Comprehensive Antibiotic Resistance Database (CARD v3.2.6) (Alcock et al., 2023). Contigs of plasmid origin were predicted using plasmid finder (v2.1.6) and plaScope (v1.3.1) (Carattoli and Hasman, 2020).

*Klebsiella pneumoniae* isolates and their related species complex phylogroups, sequence type (ST) assignment, and antibiotic resistance gene detection were performed using Kleborate (Lam et al., 2021). Plasmid replicons were identified using the PathogenWatch platform,^1^ an online global database for genomic surveillance of *K. pneumoniae* isolates (Argimón et al., 2021). *Enterobacter cloacae* isolates were analysed using Ridom SeqSphere (v10.0.2) (Jünemann et al., 2013).

### Data analysis

Categorical variables were summarized as frequencies with their percentages and compared between patients with ESBL and non-ESBL *Enterobacterales* using Fisher’s exact test.

Age was presented as mean ± standard deviation (SD) and median [interquartile range] and compared with the presence or absence of ESBL *Enterobacterales* using the student *t*-test. Results with *p* < 0.05 were considered statistically significant. Those risk factors that were statistically significant with *p* < 0.05 were included in a multivariable regression model assessing which predictors are associated with ESBL *Enterobacterales* when controlling for other factors. Odds ratios (ORs) and their 95% confidence intervals (95% CIs) of the unadjusted and adjusted models and their *p*-values were reported. Statistical analyses were performed using R (version 4.2.2) (R Core Team, 2022).

## Results

We enrolled 326 eligible patients meeting IDSA criteria for UTI. Out of these, 166 (50.9%) were outpatients, 189 (58.0%) were female, and the median age was 35.5 years [IQR: 25–52]. The collective prevalence of ESBL-PE in stool was 70.9% (231/326 patients). A total of 860 phenotypically distinct bacterial isolates were obtained from the 326 stool samples. Among these, 347 (40.3%) were ESBL-producing Enterobacterales. *E. coli* accounted for the majority of all ESBL-producing *Enterobacterales* 219 (62.8%) followed by *K. pneumoniae* 115 (33.0%), details in Table 1.

**Table 1:** ESBL *Enterobacterales* detected in stool.

ESBL bacteria	Frequency *N* = 347 *n* (%)
*E. coli*	219 (62.8%)
*K. pneumoniae*	115 (33.0%)
*E. cloacae*	5 (1.4%)
*M. morganii*	5 (1.4%)
*K. aerogenes*	1 (0.3%)
*P. vulgaris*	1 (0.3%)
*S. marcescens*	1 (0.3%)

Additionally, bacterial growth in urine was 3.7% (12 out of 326 patients). The bacterial isolates detected in urine included: four (Zamudio et al., 2024) *E. coli,* four (Zamudio et al., 2024) *P. aeruginosa*, three (Bokhary et al., 2021) *K. pneumoniae* and one (1) *P. mirabilis.* ESBL was only detected in one *E. coli* urinary isolate but was not identical to the concomitant ESBL *E. coli* detected in stool.

### Geographical distribution of ESBL *Enterobacterales* at the patient level

The distribution of ESBL-PE carriers based on their geographical origin is illustrated in Supplementary Figure S1. The figure displays the number of patients who are carriers of ESBL in different villages.

### Genomic diversity of ESBL *Enterobacterales*

**Figure 2:**
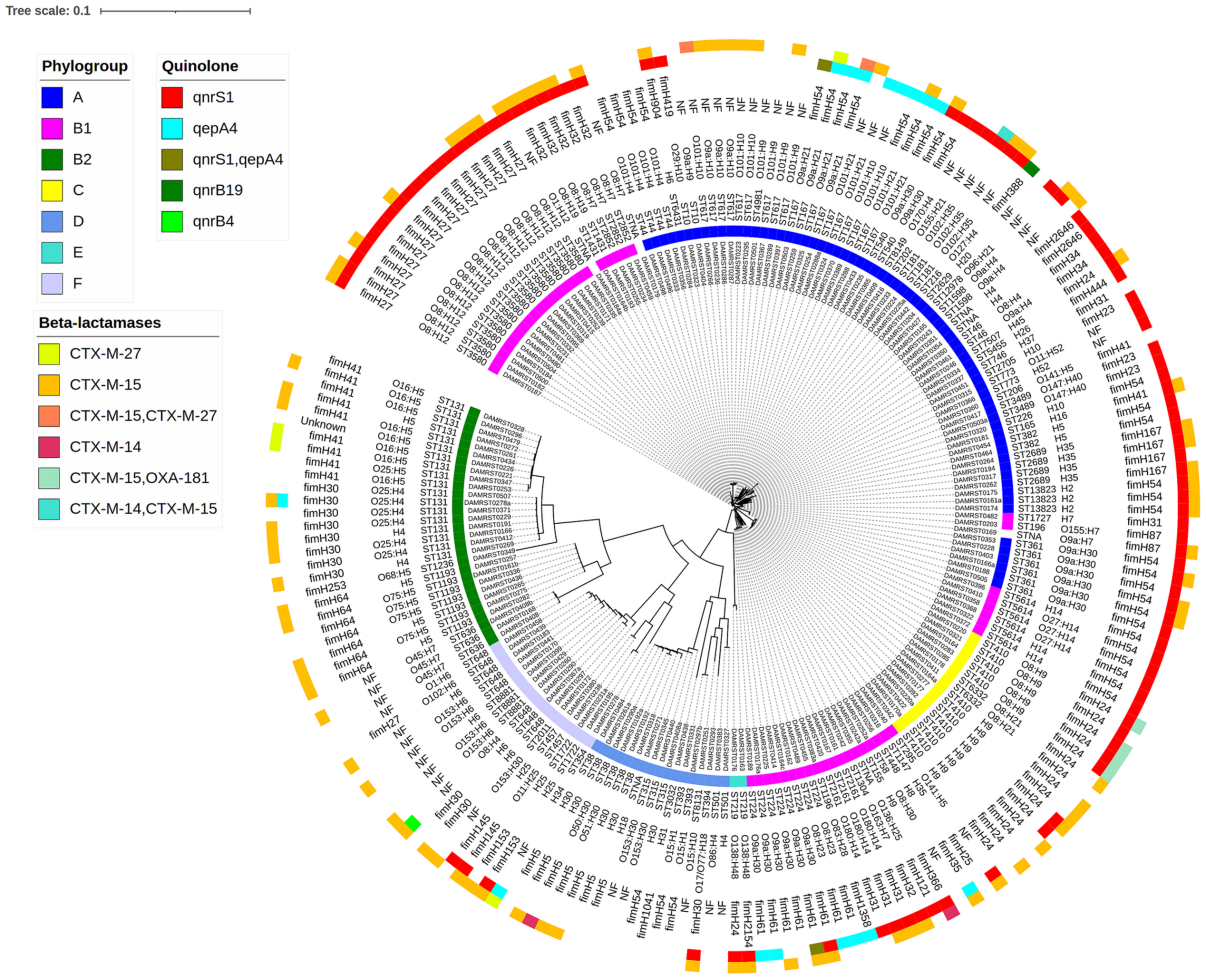
SNP-based phylogenetic tree of 219 ESBL-*E. coli*. From center to periphery, the layers correspond to the isolate name, phylogroup, sequence type (according to the Achtman scheme), serotype, the fimH allele, the quinolone resistance gene, and beta-lactamase genes.

- Genomic diversity of ESBL *E. coli* (*n* = 219, Figure 2; Supplementary Table S1)

The ESBL-*E. coli* mainly belonged to phylogroup A (79/219, 36.0%), followed by phylogroup B1 (50/219, 23.0%) and B2 (29/219, 13.0%).

Multilocus sequence typing (MLST) using the Achtmann scheme revealed 61 distinct sequence types (STs). The predominant STs identified were B2-ST131 (18/219, 8.2%), B1-ST 3580 (17/219, 7.7%), C-ST410 (14/219, 6.4%), A-ST167 (12/219, 5.5%), and A-ST 617 (12/219, 5.5%).

*In silico* serotyping revealed the presence of 76 distinct serotypes. The predominant serotypes were O8:H12 (17/219, 7.7%), O9a: H30 (15/219, 6.8%), H9 (9/219, 4.1%), and O16:H5 (8/219, 3.7%). Additionally, a total of 41 different *fimH* alleles were identified. The primary *fimH* alleles detected included *fimH27* (18/219, 8.2%), *fimH24* (17/219, 7.7%), *fimH54* (16/219, 7.3%) and *fimH30* (12/219, 5.5%).

#### Antimicrobial resistance and plasmid replicon content (Supplementary Table S2)

Among the 219 ESBL *E. coli* isolates, CTX-M-15 (189/219, 86.3%) emerged as the predominant ESBL gene, followed by CTX-M-27 (15/219, 6.8%). The quinolone resistance gene *qnrS1* was predominant among isolates (97/219, 44.3%) 0.3.7% of the *E. coli* were carbapenemase producers, all harboring OXA-181 (8/219). Plasmid analysis in the 219 ESBL-*E. coli* isolates showed diversity in replicons, with IncFIB (AP001918) being the most common (122/219, 55.7%), followed by IncFIA (74/219, 33.8%).

- Genomic diversity of ESBL *K. pneumoniae and related species* (*n* = 115, Supplementary Table S3)

Of 115 *K. pneumoniae* 90 isolates (78.2%) were identified as *K. pneumoniae /* phylogroup Kp1, (19/115, 16.5%) *Klebsiella quasipneumoniae /* phylogroup Kp2, (4/115, 3.5%) *Klebsiella* var*iicola* / Kp3 and (2/115, 1.7%) *Klebsiella quasivariicola*/Kp6. Remarkably, among 115 *K. pneumoniae* isolates, MLST using Pasteur scheme highlighted 61 different STs. The top four most common STs identified were ST 110 (6/115, 5.2%), ST 334 (6/115, 5.2%), ST 3717 (6/115, 5.2%) and ST 17 (5/115, 4.3%).

#### Antimicrobial resistance and plasmid replicon content (Supplementary Tables S4, S5)

ESBL genes were identified among 115 *K. pneumoniae* and its related species with CTX-M-15 (106/115, 92.2%) being predominant. Carbapenemase KPC-2 was detected in 1.7% (2/115). The quinolone resistance gene *qnrS1* was predominant among isolates 84 (73.0%) (84/115, 73.0%) followed by *qnrB6* (9/115, 7.8%).

Plasmid analysis showed diversity in replicons, with IncFIB (K) being the most common (95/115, 82.6%), followed by IncY (44/115, 38.3%), IncR (42/115, 36.5%) and IncFII (K) (39/115, 33.9%).

- Genomic diversity of ESBL *E. cloacae* (*n* = 5, Supplementary Table S6)

Out of five *E. cloacae*, three different ST were detected ST1, ST171, and ST 922. Four had CTX-M-15.

### Factors associated with fecal carriage of ESBL-PE

Of the 326 patients, 312 (95.7%) indicated to not have used antibiotics in the past 2 months, 317 (97.2%) had history of UTI and 324 (99.4%) had no history of diabetes.

No statistically significant associations were found between a history of UTI, history of diabetes, past antibiotic use, pregnancy, animal interaction and ESBL carriage.

Following the results of the associations presented in Table 2, the following explanatory variables were selected to include in a multivariable model assessing ESBL-PE risk: age and patient status. In the multivariable analysis, the odds of ESBL-PE carriage decreased with increasing age (OR: 0.98, 95% CI: 0.97–0.99) and were higher in inpatients (OR: 1.77, 95% CI: 1.08–2.91) than in outpatients (Table 3).

**Table 2:** Bivariable analysis of risk factors for ESBL carriage.

Variable	ESBL negative (*N* = 95) *n* (%)	ESBL positive (*N* = 231) *n* (%)	Total (*N* = 326) *n* (%)	*p*-value
Age				
Mean (SD)	43.8 (18.4)	37.4 (16.8)	39.3 (17.5)	
Median [Min, Max]	40.0 [18.0, 86.0]	33.0 [18.0, 99.0]	35.5 [18.0, 99.0]	0.004
IQR [25th, 75th]	[29.0, 58.0]	[24.0, 48.0]	[25.0, 52.0]	
Gender				
Female	52 (54.7%)	137 (59.3%)	189 (58.0%)	0.461
Male	43 (45.3%)	94 (40.7%)	137 (42.0%)	
Patient status				
Inpatient	37 (38.9%)	123 (53.2%)	160 (49.1%)	0.021
Outpatient	58 (61.1%)	108 (46.8%)	166 (50.9%)	
Past antibiotic use (2 months)				
No	92 (96.8%)	220 (95.2%)	312 (95.7%)	0.765
Yes	3 (3.2%)	11 (4.8%)	14 (4.3%)	
Pregnant				
No	48 (50.5%)	120 (51.9%)	168 (51.5%)	0.445
Yes	4 (4.2%)	17 (7.4%)	21 (6.4%)	
UTI history				
No	2 (2.1%)	5 (2.2%)	7 (2.1%)	0.999
Yes	93 (97.9%)	224 (97.0%)	317 (97.2%)	
Diabetes history				
No	94 (98.9%)	230 (99.6%)	324 (99.4%)	0.292
Yes	1 (1.1%)	0 (0%)	1 (0.3%)	
Contact with cats				
No	65 (68.4%)	160 (69.3%)	225 (69.0%)	0.875
Yes	19 (20.0%)	43 (18.6%)	62 (19.0%)	
Contact with dogs				
No	59 (62.1%)	140 (60.6%)	199 (61.0%)	0.778
Yes	24 (25.3%)	63 (27.3%)	87 (26.7%)	
Contact with cattle				
No	64 (67.4%)	140 (60.6%)	204 (62.6%)	0.253
Yes	20 (21.1%)	63 (27.3%)	83 (25.5%)	
Contact with chickens				
No	44 (46.3%)	96 (41.6%)	140 (42.9%)	0.440
Yes	40 (42.1%)	107 (46.3%)	147 (45.1%)	
Other animal contact				
No	64 (67.4%)	138 (59.7%)	202 (62.0%)	0.360
Yes	17 (17.9%)	50 (21.6%)	67 (20.6%)	

**Table 3:** Multivariable analysis of risk factors for ESBL carriage.

Predictors of ESBL	Unadjusted ORs	95% CIs	*p*-value	Adjusted ORs	95% CIs	*p*-value
Age	0.980	0.967–0.993	0.0030	0.980	0.967–0.993	0.0239
Patient status (Inpatient)	1.785	1.101–2.921	0.0196	1.765	1.082–2.907	0.0036

## Discussion

This study found that more than two-third (70.9%) of patients presenting with UTI were ESBL-PE carriers. This high prevalence of carriage in rural Tanzania serves as an important indicator of antibiotic resistance in the area. Prevalence rates for ESBL-PE colonization vary substantially among different populations in Tanzania (Manyahi et al., 2020). Geographical distribution data revealed potential hotspots of ESBL-PE carriage rates across villages, with some showing higher prevalence. These findings highlight areas with potentially elevated transmission risk, which could guide future studies and inform targeted intervention strategies.

Previous studies have reported lower carriage rates in the urban area of Dar es Salaam, with rates of 32.6% in HIV patients and 34.3% in children (Manyahi et al., 2020; Tellevik et al., 2016). Additionally, a rate of 59.7% has been observed in adult patients (Kibwana et al., 2020). In contrast, other research has found much higher (91.5%) colonization rates among hotel employees on the island of Zanzibar (Büdel et al., 2019). Furthermore, most studies have linked risk factors such as sharing of toilets (Erb et al., 2018), poor working environments and hygienic practices (Mwanginde et al., 2021), use of third-generation cephalosporins (Manyahi et al., 2020), history of antibiotic use (Moremi et al., 2018), old age (Kibwana et al., 2020) with increased ESBL fecal carriage.

These findings highlight the diverse distribution of ESBL-PE colonization within Tanzania, suggesting potential differences between urban and rural settings, as well as among various demographic groups.

Notably, this study highlights that younger individuals and inpatients were associated with an increased risk of being ESBL carriers. Inpatients exhibited a higher ESBL carriage rate than outpatients, consistent with literature identifying prolonged hospitalizations and extensive antibiotic treatments as key predictors for ESBL carriage (Mai and Espinoza, 2023; Moremi et al., 2021).

Our study did not reveal significant associations between ESBL carriage and factors such as previous antibiotic use, or animal contact. However, patients might not have reported previous antibiotic use on the one hand, on the other hand the relatively small sample size limits our ability to draw definitive conclusions. Further research with larger cohorts may be necessary to fully elucidate the relationships between these variables and ESBL carriage in this population.

Despite the high prevalence of patients with a history of UTI (97.2%), our study did not find an association between this factor and ESBL carriage. This disparity can be attributed to most individuals delaying seeking medical treatment, which may result in recurrent UTIs. Furthermore, patients frequently self-medicate using over-the-counter antibiotics (Torres et al., 2021; Maldonado-Barragán et al., 2024) or use traditional remedies, such as herbal medicines. This practice often results in a common history of UTIs among those who do not seek medical attention. In addition, in rural settings, poor sanitation, limited access to clean water, and inadequate hygiene infrastructure increase the risk of UTIs and their recurrence (Mwambete and Msigwa, 2017). We understand that the 97.2% figure might differ from patient populations in other settings. However, this difference highlights the challenges faced in rural health care due to the factors outlined earlier.

ESBL-producing *E. coli* emerged as the dominant colonizing species, followed by *K. pneumoniae,* with the CTX-M-15 enzyme being the most prevalent ESBL. The ubiquity of CTX-M-15 genes in both *E. coli* (86.3%) and *K. pneumoniae* (92.2%) aligns with global trends as well as with studies from Tanzania (Bevan et al., 2017; Moremi et al., 2017; Onduru et al., 2021; Slown et al., 2022; Sonda et al., 2018). Highly mobile genetic elements are responsible for carrying the predominant CTX-M genes between bacterial strains. The scenario is further supported by the established association between CTX-M enzymes and *E. coli* IncF resident plasmids, which are particularly well-adapted to *E. coli* strains and can readily transfer between them (Woerther et al., 2013).

Carbapenemases were only detected in a minority of cases, such as OXA-181 in 3.7% of *E. coli* and KPC-2 in 1.7% of *K. pneumoniae*. This low prevalence of carbapenemases suggests that carbapenems are likely to remain an effective treatment option for the time being. However, continued vigilance is essential to uphold infection control measures and prevent the potential rise of carbapenem-resistant isolates in our setting. We observed a phylogenetic diversity among the ESBL-producing *E. coli*, with the majority belonging to phylogenetic group A, followed by B1 and B2. ST-131 was the most common sequence type in the latter phylogroup. This finding aligns with earlier studies that have shown considerable phylogenetic diversity within ESBL-producing *E. coli* populations (Bevan et al., 2017). We also observed a notable diversity in sequence types, serotypes, and *fimH* alleles.

The diverse phylogroups and sequence types observed among ESBL-PE isolates, suggest complex transmission dynamics. Our research underscores the need for further research into community transmission patterns in resource-limited settings.

Limitations of the study include reliance on short-read sequencing data and the lack of environmental sampling, which restricted the comprehensive investigation of plasmid population structures and environmental transmission factors.

In addition, we were not able to investigate the relationship between fecal ESBL carriage and urinary tract infections, as most urinary cultures remained negative or showed only insignificant growth. The negative urine cultures could be due to (a) some symptoms may have been caused by conditions other than a UTI, such as sexually transmitted infections (STIs) which cannot be diagnosed by urine cultures, (b) medical incompliance, patients might be consuming antibiotics but hide this information from the clinician these consumed antibiotic agents may inhibit in-vitro culture growth of bacteria in the urine in this study we did not perform any inhibitory test to detect the presence of any previous antibiotic therapy in our urine samples (c) there may have been improper collection, handling and processing of urine samples in our setting. Our results challenge the assumption that all patients presenting with UTI symptoms will have positive urine cultures but it underscores the importance of including differential diagnoses in patients presenting with UTI-like symptoms. In future, further studies are needed to investigate the causes of these symptoms and the role of non-UTI infections and antibiotic stewardship in our settings. Without correlating fecal ESBL carriage to urine culture results, we cannot definitively establish the gut as a reservoir for invasive UTIs in this population.

In this study, we observed a low rate of history of antibiotic use. This might be because certain patients were unaware/ cannot accurately recall when last they used antibiotics. Additionally, we did not capture self-treatment with non-prescribed antibiotics or the use of traditional remedies in our setting. We recognize the potential for recall bias, given the reliance on self-reported information.

We recognize other ESBL risk factors such as history of surgery, hospitalization in the last 6 months, urinary catheterization, history of ESBL colonization, travel to other countries, and number of people living in the same house are under-recorded. In this study, due to limited access to patient records, we could not have details for these risk factors. Additionally, some factors, such as international travel history, may have been less applicable in our study population compared to urban settings.

Our study included all patients who met UTI IDSA criteria, which introduces selection bias and explains why we had a high proportion of patients with a history of UTI. The model selection for our multivariable model was prone to bias given that we used a *p*-value cutoff from the bivariate associations to determine which variables to include, however, this was chosen due to limited data availability and unbalanced groupings for other categorical factors. Statistical significance should be taken with caution due to the observational nature of the study.

In conclusion, this study reveals a high fecal carriage rate of ESBL Enterobacterales, especially among younger individuals in Tanzania, underscoring the need to investigate transmission dynamics beyond hospital settings. Although only one ESBL-positive isolate was identified as a cause of UTI, the high fecal carriage rate indicates a significant potential for infections with resistant organisms. This suggests a broader reservoir of resistant pathogens, particularly in areas with limited infection control measures.

Our findings stress the importance of understanding local epidemiology and highlight the need for healthcare interventions in rural areas to ensure proper treatments, appropriate antibiotic use, and education on personal hygiene. These results have significant implications for empirical treatment strategies and infection prevention policies in similar contexts.

## Data Availability

The datasets presented in this study can be found in online repositories. The names of the repository/repositories and accession number(s) can be found in the article/Supplementary material.

## Footnotes

^1^ https://pathogen.watch
