# Supplementary figures and images for: High prevalence of fecal carriage of extended-spectrum beta-lactamase producing Enterobacterales among patients with urinary tract infections in rural Tanzania
Source: Front Microbiol. 2025 Jan 6;15:1517182. doi: 10.3389/fmicb.2024.1517182 (PMC11743186; doi:10.3389/fmicb.2024.1517182)

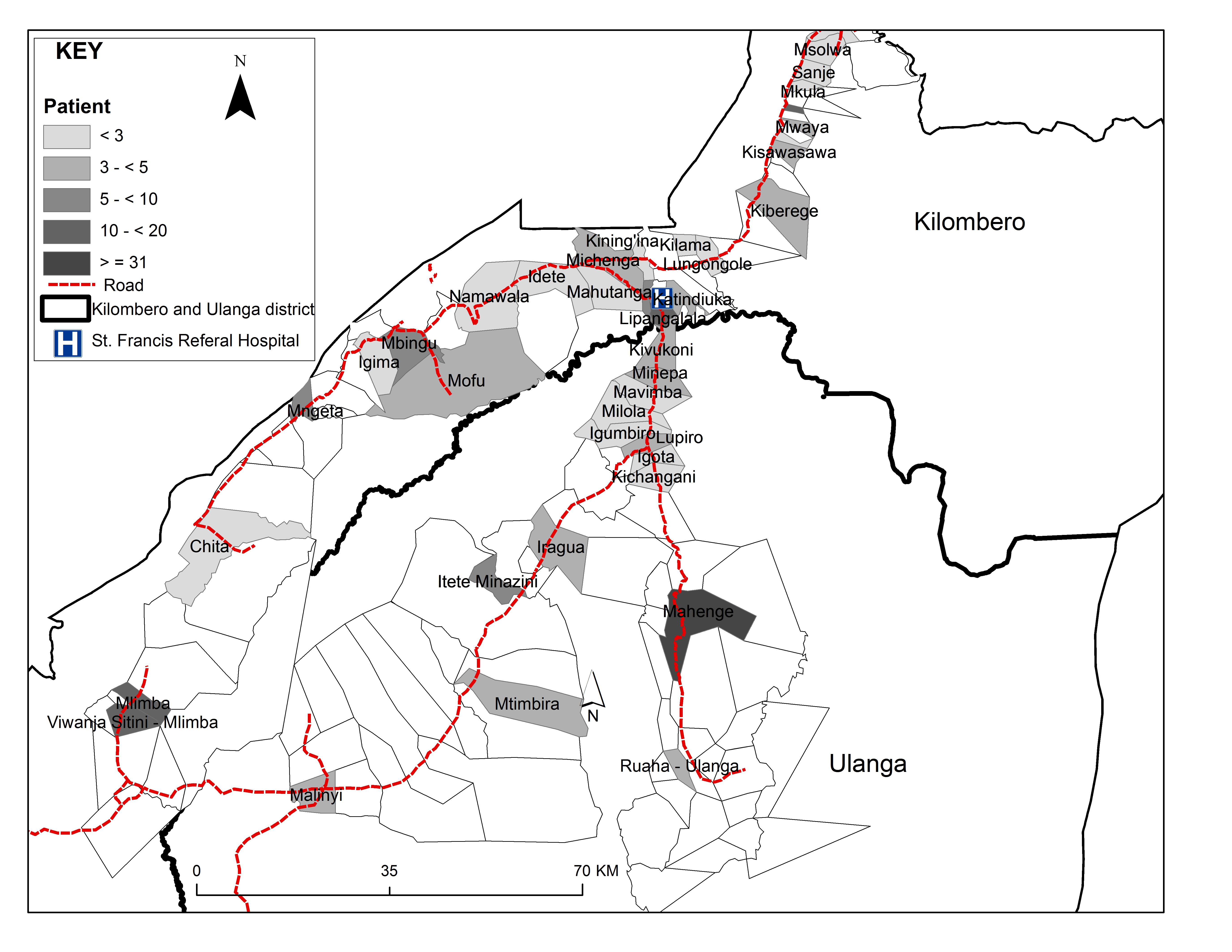

Supplement: Supplementary file 1 [file Image_1.JPEG]
