# Supplementary material for: High prevalence of fecal carriage of extended-spectrum beta-lactamase producing Enterobacterales among patients with urinary tract infections in rural Tanzania
Source: Front Microbiol. 2025 Jan 6;15:1517182. doi: 10.3389/fmicb.2024.1517182 (PMC11743186; doi:10.3389/fmicb.2024.1517182)
Supplement: Supplementary file 2 [file Table_1.DOCX]

**Table S1** ESBL-*E. coli* (n = 219) molecular patterns

| **Phylogroup** | **ST** | **Serotype** | **fimH allele** | **Beta-lactamase Typing** |
| --- | --- | --- | --- | --- |
| A (79) | ST10 (2) | H10:O29 (1) | fimH419 (1) | **CTX-M-15** |
|  |  | H9:O9a (1) | NF (1) | **CTX-M-15**, OXA-1 |
|  | ST12978 (1) | H21:O96 (1) | NF (1) | TEM-234, TEM-217, TEM-198, TEM-104, TEM-1, **CTX-M-15** |
|  | ST13823 (3) | H2 (3) | fimH54 (1) | **CTX-M-15** |
|  | ST1598 (2) | H4:O9a (2) | NF (2) | TEM-169, **CTX-M-15** |
|  |  |  |  | TEM-1, **CTX-M-15** |
|  | ST165 (1) | H16 (1) | fimH41 (1) | TEM-122, **CTX-M-15** |
|  | ST167 (12) | H10:O101 (4) | fimH54 (2) | TEM-1, CTX-M-27 |
|  |  |  |  | TEM-1, CTX-M-27 |
|  |  |  | NF (2) | CTX-M-27 (2) |
|  |  | H21:O101 (4) | fimH54 (2) | **CTX-M-15** |
|  |  |  | NF (2) | TEM-1, **CTX-M-15** (2) |
|  |  | H21:O9a (3) | fimH54 (3) | TEM-190, **CTX-M-27** |
|  |  |  |  | **CTX-M-15**, CTX-M-27 |
|  |  |  |  | CTX-M-27 |
|  | ST181 (3) | H35:O102 (3) | NF (3) | CTX-M-14, **CTX-M-15** |
|  |  |  |  | **CTX-M-15** (2) |
|  | ST202 (1) | H21:O155 (1) | NF (1) | **CTX-M-15** |
|  | ST206 (1) | H5:O141 (1) | fimH41 (1) | CTX-M-102 |
|  | ST226 (1) | H10 (1) | fimH54 (1) | **CTX-M-15** |
|  | ST2629 (1) | H20 (1) | NF (1) | **CTX-M-15** |
|  | ST2689 (6) | H35 (6) | fimH167 (6) | **CTX-M-15** (6) |
|  | ST2705 (2) | H10 (2) | fimH23 (2) | TEM-1, **CTX-M-15** (2) |
|  | ST3489 (2) | H40:O147 (2) | fimH23 (2) | **CTX-M-15** |
|  | ST361 (6) | H30:O9a (6) | fimH54 (6) | TEM-1 |
|  |  |  |  | TEM-1, **CTX-M-15** (5) |
|  | ST382 (2) | H5 (2) | fimH54 (2) | TEM-1, **CTX-M-15** (2) |
|  | ST44 (4) | H4:O101 (4) | fimH54 (4) | **CTX-M-15**, OXA-1 (3) |
|  |  |  |  | **CTX-M-15** |
|  | ST46 (2) | H4:O8 (1) | fimH34 (1) | TEM-1, **CTX-M-15** |
|  |  | H4:O9a (1) | fimH34 (1) | TEM-1, **CTX-M-15** |
|  | ST4981 (1) | H9:O101 (1) | NF (1) | TEM-1, **CTX-M-15**, OXA-1 |
|  | ST540 (2) | H30:O9a (2) | fimH54 (2) | TEM-1, **CTX-M-15** (2) |
|  | ST5455 (1) | H26 (1) | fimH444 (1) | TEM-1, **CTX-M-15** |
|  | ST617 (12) | H10:O101 (4) | NF (4) | **CTX-M-15**, OXA-1 (3) |
|  |  |  |  | TEM-1, **CTX-M-15,** CTX-M-27, OXA-1 |
|  |  | H10:O9a (5) | NF (5) | TEM-1, **CTX-M-15,** OXA-320 (5) |
|  |  | H9:O101 (3) | NF (3) | TEM-1, **CTX-M-15** (3) |
|  | ST6431 (1) | H6 (1) | fimH904 (1) | TEM-122, **CTX-M-15** |
|  | ST746 (2) | H37 (2) | fimH31 (2) | TEM-1, **CTX-M-15** (2) |
|  | ST7507 (1) | H45 (1) | fimH24 (1) | TEM-117, **CTX-M-15** |
|  | ST773 (2) | H52 (1) | NF (1) | **CTX-M-15** |
|  |  | H52:O11 (1) | NF (1) | **CTX-M-15** |
|  | ST8149 (1) | H4:O170 (1) | NF (1) | TEM-1, **CTX-M-15** |
|  | ST216 (1) | H4:O127 (1) | fimH388 (1) | **CTX-M-15** |
|  | NA (3) | H4 (3) | fimH2646 (3) | TEM-1, CTX-M-55 (3) |
| B1 (50) | ST1196 (1) | H28:O83 (1) | fimH1358 (1) | CTX-M-27 |
|  | ST1304 (1) | H7:O163 (1) | fimH32 (1) | TEM-117,**CTX-M-15** |
|  | ST1431 (2) | H19:O8 (2) | fimH32 (2) | TEM-1, **CTX-M-15** (2) |
|  | ST1727 (1) | H7 (1) | fimH31 (1) | TEM-1, **CTX-M-15** |
|  | ST196 (1) | H7:O155 (1) | fimH87 (1) | CTX-M-55 |
|  | ST2161 (4) | H14:O180 (4) | fimH31 (4) | **CTX-M-15** (4) |
|  | ST224 (9) | H23:O8 (2) | fimH61 (2) | CTX-M-27 (2) |
|  |  | H30:O9a (7) | fimH61 (7) | TEM-1, **CTX-M-15** (3) |
|  |  |  |  | TEM-169, **CTX-M-15** (3) |
|  |  |  |  | TEM-33, TEM-169, **CTX-M-15** |
|  | ST2852 (3) | H7:O8 (3) | fimH32 (3) | TEM-1, **CTX-M-15** (3) |
|  | ST295 (1) | H5:O141 (1) | NF (1) | **CTX-M-15** |
|  | ST3580 (17) | H12:O8 (17) | fimH27 (17) | **CTX-M-15** (17) |
|  | ST448 (1) | H8 (1) | fimH35 (1) | **CTX-M-15** |
|  | ST5614 (6) | H14 (3) | fimH54 (3) | **CTX-M-15** (3) |
|  |  | H14:O27 (3) | fimH54 (3) | **CTX-M-15** (3) |
|  | ST155 (1) | H9 (1) | fimH366 (1) | TEM-1, CTX-M-14 |
|  | ST1147 (1) | H35 (1) | fimH25 (1) | **CTX-M-15** |
|  | NA (1) | H25:O136 (1) | fimH121 (1) | **CTX-M-15** |
| B2 (29) | ST1193 (7) | H5 (3) | fimH64 (3) | **CTX-M-15**, OXA-1 |
|  |  |  |  | TEM-1,**CTX-M-15** (2) |
|  |  | H5:O75 (4) | fimH64 (4) | **CTX-M-15**, OXA-1 (4) |
|  | ST131 (18) | H4 (2) | fimH30 (2) | TEM-1, **CTX-M-15**, OXA-1 |
|  |  |  |  | **CTX-M-15**, OXA-1 |
|  |  | H4:O25 (6) | fimH30 (6) | TEM-1, **CTX-M-15**, OXA-1 (4) |
|  |  |  |  | **CTX-M**-15, OXA-1 |
|  |  |  |  | **CTX-M-15** |
|  |  | H5 (1) | fimH41 (1) | TEM-1, **CTX-M-15** |
|  |  | H5:O16 (1) | NF (1) | CTX-M-27 |
|  |  | H5:O16 (7) | fimH41 (7) | **CTX-M-15** (3) |
|  |  |  |  | CTX-M-27 (2) |
|  |  |  |  | TEM-1, **CTX-M-15** (2) |
|  |  | H5:O25 (1) | fimH41 (1) | TEM-1, **CTX-M-15** |
|  | ST636 (3) | H7:O45 (3) | NF (3) | TEM-1, **CTX-M-15** |
|  |  |  |  | TEM-1, **CTX-M-15** |
|  |  |  |  | TEM-1, **CTX-M-15** |
|  | ST1236 (1) | H5:O68 (1) | fimH253 (1) | **CTX-M-15** |
| C (16) | ST410 (14) | H9 (8) | fimH24 (8) | **CTX-M-15**, OXA-1 (4) |
|  |  |  |  | TEM-1, **CTX-M-15**, OXA-1, OXA-181 |
|  |  |  |  | **CTX-M-15** (3) |
|  |  | H9:O8 (6) | fimH24 (6) | TEM-1, **CTX-M-15**, OXA-1, OXA-181 (5) |
|  |  |  |  | OXA-181 |
|  | ST6332 (2) | H21:O8 (2) | fimH24 (2) | TEM-1, **CTX-M-15**, OXA-1 (2) |
| D (16) | ST3032 (1) | H31 | fimH1041 (1) | TEM-1, **CTX-M-15** |
|  | ST315 (3) | H30 (1) | fimH54 (1) | TEM-1, **CTX-M-15** |
|  |  | H30:O153 (2) | NF (2) | TEM-1, **CTX-M-15** (2) |
|  | ST38 (6) | H30 (4) | fimH5 (4) | TEM-1, **CTX-M-15**, OXA-1 |
|  |  |  |  | **CTX-M-15** (2) |
|  |  |  |  | TEM-1, CTX-M-14 |
|  |  | H30:O50 (1) | fimH5 (1) | TEM-1, CTX-M-14 |
|  |  | H30:O51 (1) | fimH5 (1) | TEM-1, **CTX-M-15**, OXA-1, PAU-1 |
|  | ST393 (2) | H1:O15 (2) | fimH54 (2) | **CTX-M-15** |
|  |  |  |  | **CTX-M-15** |
|  | ST394 (1) | H18:O17/O77 (1) | fimH30 (1) | TEM-117, **CTX-M-15** |
|  | ST501 (2) | H4 (1) | NF (1) | **CTX-M-15** |
|  |  | H4:O86 (1) | NF (1) | **CTX-M-15** |
|  | NA (1) | H18 (1) | fimH5 (1) | TEM-1, **CTX-M-15** |
| E (2) | ST219 (2) | H48:O138 (2) | fimH24 (1) | TEM-122, **CTX-M-15** |
|  |  |  | fimH2154 (1) | **CTX-M-15** |
| F (19) | ST1722 (2) | H25 (2) | fimH153 (2) | TEM-1, **CTX-M-15** (2) |
|  | ST2011 (1) | H30:O153 (1) | NF (1) | TEM-1, **CTX-M-15** |
|  | ST457 (2) | H25 (1) | fimH145 (1) | **CTX-M-15** |
|  |  | H25:O11 (1) | fimH145 (1) | TEM-135, **CTX-M-15** |
|  | ST648 (9) | H4:O8 (1) | NF (1) | TEM-1, **CTX-M-15**, OXA-1 |
|  |  | H6 (3) | fimH30 (3) | **CTX-M-15**, OXA-1 (3) |
|  |  | H6 (1) | NF (1) | **CTX-M-15** |
|  |  | H6:O1 (1) | NF (1) | **CTX-M-15**, OXA-1 |
|  |  | H6:O102 (1) | fimH27 (1) | TEM-1, **CTX-M-15**, OXA-1 |
|  |  | H6:O153 (2) | NF (2) | TEM-1, **CTX-M-15**, OXA-1 (2) |
|  | ST8881 (4) | H6 (2) | NF (2) | TEM-1, **CTX-M-15**, OXA-1 |
|  |  |  |  | TEM-1, **CTX-M-15** |
|  |  | H6:O153 (2) | NF (2) | TEM-1, **CTX-M-15**, OXA-1 (2) |
|  | ST354 (1) | H34 (1) | NF (1) | TEM-1, CTX-M-27, OXA-1 |
| Unknown (8) | NA (1) | NA (1) | Unknown (1) | SHV-11 |
|  | NA (7) | H12:O1 (1) | NF (1) | **CTX-M-15** |
|  |  | H7:O8 (1) | NF (1) | **CTX-M-15**, OXA-1, TEM-1 |
|  |  | H7:O9a (1) | fimH87 (1) | TEM-1, CTX-M-226 |
