# Supplementary material for: High prevalence of fecal carriage of extended-spectrum beta-lactamase producing Enterobacterales among patients with urinary tract infections in rural Tanzania
Source: Front Microbiol. 2025 Jan 6;15:1517182. doi: 10.3389/fmicb.2024.1517182 (PMC11743186; doi:10.3389/fmicb.2024.1517182)
Supplement: Supplementary file 3 [file Table_2.DOCX]

**Table S2** Plasmid replicons of 219 *ESBL E. coli* isolates

| **Classification** | **Replicon type** | **Frequency (n, %). N = 219** |
| --- | --- | --- |
| IncF plasmids | IncFIB(AP001918) | 122 (55.7%) |
|  | IncFIA | 74 (33.8%) |
|  | IncFII(pRSB107) | 40 (18.3%) |
|  | IncFII(pAMA1167-NDM-5) | 27 (12.3%) |
|  | IncFIB(K) | 16 (7.3%) |
|  | IncFII(29) | 13 (5.9%) |
|  | IncFIA(HI1) | 7 (3.2%) |
|  | IncFIB(pB171) | 6 (2.7%) |
|  | IncFII(pHN7A8) | 4 (1.8%) |
|  | IncFII(pSE11) | 1 (0.5%) |
| IncH plasmids | IncHI1B | 3 (1.4%) |
|  | IncHI2 | 2 (0.9%) |
|  | IncHI2A | 2 (0.9%) |
|  | IncHI1A | 2 (0.9%) |
|  | IncHI1B(R27) | 2 (0.9%) |
| IncI plasmids | IncI1 | 16 (7.3%) |
|  | IncI2 | 5 (2.3%) |
|  | IncB/O/K/Z | 11 (5.0%) |
|  | p0111 | 1 (0.5%) |
| IncX plasmids | IncX1 | 14 (6.4%) |
|  | IncX4 | 5 (2.3%) |
| IncY plasmids | IncY | 41 (18.7%) |
| IncR plasmids | IncR | 3 (1.4%) |
| Col plasmids | Col156 | 41 (18.7%) |
|  | Col(BS512) | 28 (12.8%) |
|  | Col(MG828) | 14 (6.4%) |
|  | Col440I | 7 (3.2%) |
|  | Col440II | 4 (1.8%) |
|  | ColRNAI | 4 (1.8%) |
|  | Col(KPHS6) | 3 (1.4%) |
|  | Col8282 | 2 (0.9%) |
| Unknown | NA | 20 (9.1%) |

NA = Not Applicable
