# Supplementary material for: High prevalence of fecal carriage of extended-spectrum beta-lactamase producing Enterobacterales among patients with urinary tract infections in rural Tanzania
Source: Front Microbiol. 2025 Jan 6;15:1517182. doi: 10.3389/fmicb.2024.1517182 (PMC11743186; doi:10.3389/fmicb.2024.1517182)
Supplement: Supplementary file 4 [file Table_3.DOCX]

**Table S3** ESBL K. pneumoniae and its related species phylogroups and sequence types

| **Species /Phylogroups** | **Sequence Type (ST)** | **Frequency (n, %) N= 115** |
| --- | --- | --- |
| *Klebsiella pneumoniae* / Kp1 | 110 | 6 (5.2%) |
|  | 3717 | 6 (5.2%) |
|  | 17 | 5 (4.3%) |
|  | 37 | 5 (4.3%) |
|  | 45 | 4 (3.5%) |
|  | 185 | 3 (2.6%) |
|  | 307 | 3 (2.6%) |
|  | 3113 | 3 (2.6%) |
|  | 313 | 3 (2.6%) |
|  | 35 | 3 (2.6%) |
|  | 2938 | 2 (1.7%) |
|  | 7481 | 2 (1.7%) |
|  | 147 | 2 (1.7%) |
|  | 189 | 2 (1.7%) |
|  | 258 | 2 (1.7%) |
|  | 337 | 2 (1.7%) |
|  | 34 | 2 (1.7%) |
|  | 39 | 2 (1.7%) |
|  | 6516 | 2 (1.7%) |
|  | 896 | 2 (1.7%) |
|  | 152 | 1 (0.9%) |
|  | 292 | 1 (0.9%) |
|  | 7480 | 1 (0.9%) |
|  | 7482 | 1 (0.9%) |
|  | 7516 | 1 (0.9%) |
|  | 1296 | 1 (0.9%) |
|  | 348 | 1 (0.9%) |
|  | 5738 | 1 (0.9%) |
|  | 336 | 1 (0.9%) |
|  | 4733 | 1 (0.9%) |
|  | 3157 | 1 (0.9%) |
|  | 1263 | 1 (0.9%) |
|  | 1412 | 1 (0.9%) |
|  | 1741 | 1 (0.9%) |
|  | 1808 | 1 (0.9%) |
|  | 20 | 1 (0.9%) |
|  | 2668 | 1 (0.9%) |
|  | 2715 | 1 (0.9%) |
|  | 29 | 1 (0.9%) |
|  | 3586 | 1 (0.9%) |
|  | 36 | 1 (0.9%) |
|  | 469 | 1 (0.9%) |
|  | 482 | 1 (0.9%) |
|  | 4961 | 1 (0.9%) |
|  | 629 | 1 (0.9%) |
|  | 664 | 1 (0.9%) |
|  | 7337 | 1 (0.9%) |
|  | 967 | 1 (0.9%) |
|  | Unknown | 1 (0.9%) |
| *Klebsiella quasipneumoniae* / Kp2 | 334 | 6 (5.2%) |
|  | 367 | 3 (2.6%) |
|  | 1308 | 2 (1.7%) |
|  | 7483 | 1 (0.9%) |
|  | 138 | 1 (0.9%) |
|  | 1584 | 1 (0.9%) |
|  | 2558 | 1 (0.9%) |
|  | 3013 | 1 (0.9%) |
|  | 4218 | 1 (0.9%) |
|  | 5716 | 1 (0.9%) |
|  | Unknown | 1 (0.9%) |
| *Klebsiella variicola* / Kp3 | 771 | 4 (3.5%) |
| *Klebsiella quasivariicola* / Kp6 | 3897 | 1 (0.9%) |
|  | 2830 | 1 (0.9%) |
