# Supplementary material for: High prevalence of fecal carriage of extended-spectrum beta-lactamase producing Enterobacterales among patients with urinary tract infections in rural Tanzania
Source: Front Microbiol. 2025 Jan 6;15:1517182. doi: 10.3389/fmicb.2024.1517182 (PMC11743186; doi:10.3389/fmicb.2024.1517182)
Supplement: Supplementary file 5 [file Table_4.DOCX]

**Table S4** Antimicrobial resistance genes in ESBL-*K. pneumoniae* and its related species

| **Classification** | **Resistance gene** | **Frequency (n, %) N= 115** |
| --- | --- | --- |
| Narrow & Broad-spectrum beta-lactamase | TEM-1 | 91 (79.1%) |
|  | TEM-122 | 2 (1.7%) |
|  | TEM-163 | 2 (1.7%) |
|  | TEM-168 | 2 (1.7%) |
|  | TEM-71 | 1 (0.9%) |
|  | OKP-B-1 | 1 (0.9%) |
|  | OKP-B-17 | 6 (5.2%) |
|  | OKP-B-15 | 4 (3.5%) |
|  | OKP-B-6 | 4 (3.5%) |
|  | OKP-B-5 | 2 (1.7%) |
|  | OKP-B-3 | 1 (0.9%) |
|  | OKP-B-10 | 1 (0.9%) |
|  | OKP-B-14 | 1 (0.9%) |
|  | OKP-B-16 | 1 (0.9%) |
|  | OKP-B-2 | 2 (1.7%) |
|  | OKP-B-34 | 1 (0.9%) |
|  | OKP-B-8 | 1 (0.9%) |
|  | OKP-D-1 | 1 (0.9%) |
|  | SHV-11 | 25 (21.7%) |
|  | SHV-40 | 10 (8.7%) |
|  | OXA-1 | 10 (8.7%) |
|  | OXA-9 | 2 (1.7%) |
|  | OXA-260 | 1 (0.9%) |
|  | OXA-480 | 1 (0.9%) |
|  | OXA-508 | 1 (0.9%) |
|  | OXA-65 | 1 (0.9%) |
|  | OXA-66 | 1 (0.9%) |
|  | OXA-76 | 1 (0.9%) |
| Extended-spectrum beta-lactamase | CTX-M-15 | 106 (92.2%) |
|  | CTX-M-14 | 2 (1.7%) |
|  | SHV-14 | 1 (0.9%) |
|  | SHV-11 | 25 (21.7%) |
|  | SHV-26 | 12 (10.4%) |
|  | SHV-78 | 12 (10.4%) |
|  | SHV-145 | 12 (10.4%) |
|  | SHV-179 | 12 (10.4%) |
|  | SHV-187 | 11 (9.6%) |
|  | SHV-98 | 12 (10.4%) |
|  | SHV-194 | 11 (9.6%) |
|  | SHV-199 | 12 (10.4%) |
|  | SHV-56 | 10 (8.7%) |
|  | SHV-79 | 10 (8.7%) |
|  | SHV-85 | 10 (8.7%) |
|  | SHV-89 | 10 (8.7%) |
|  | SHV-40 | 10 (8.7%) |
|  | SHV-110 | 6 (5.2%) |
|  | SHV-81 | 5 (4.3%) |
|  | SHV-94 | 4 (3.5%) |
|  | SHV-96 | 4 (3.5%) |
|  | SHV-172 | 4 (3.5%) |
|  | SHV-111 | 3 (2.6%) |
|  | SHV-33 | 3 (2.6%) |
|  | SHV-28 | 3 (2.6%) |
|  | SHV-106 | 2 (1.7%) |
|  | SHV-13 | 2 (1.7%) |
|  | SHV-27 | 2 (1.7%) |
|  | SHV-133 | 2 (1.7%) |
|  | SHV-158 | 2 (1.7%) |
|  | SHV-159 | 2 (1.7%) |
|  | SHV-182 | 2 (1.7%) |
|  | SHV-186 | 2 (1.7%) |
|  | SHV-48 | 2 (1.7%) |
|  | SHV-67 | 2 (1.7%) |
|  | SHV-70 | 2 (1.7%) |
|  | SHV-80 | 2 (1.7%) |
|  | SHV-12 | 1 (0.9%) |
|  | SHV-38 | 1 (0.9%) |
|  | SHV-14 | 1 (0.9%) |
|  | SHV-93 | 1 (0.9%) |
|  | SHV-108 | 1 (0.9%) |
|  | SHV-168 | 1 (0.9%) |
|  | SHV-191 | 1 (0.9%) |
|  | SHV-198 | 1 (0.9%) |
|  | SHV-217 | 1 (0.9%) |
| Carbapenemase | KPC-2 | 2 (1.7%) |
| Quinolone resistance | qnrS1 | 84 (73.0%) |
|  | qnrB6 | 9 (7.8 %) |
|  | qnrB1 | 7 (6.1%) |
| Aminoglycoside modifying enzymes | aph(6)-Id | 72 (62.6%) |
|  | aph(3'')-Ib | 64 (55.6%) |
|  | aadA16 | 12 (10.4%) |
|  | aac(3)-IId | 9 (7.8 %) |
|  | aph (3')-Ia | 5 (4.3%) |
|  | aac(3)-IIa | 4 (3.5%) |
|  | aac(3)-IIe | 4 (3.5%) |
|  | aac(6')-Ib | 2 (1.7%) |
|  | aadA1 | 1 (0.9%) |
|  | aadA2b | 1 (0.9%) |
|  | ant(2'')-Ia | 1 (0.9%) |
| Fluoroquinolone and aminoglycoside | aac(6')-Ib-cr5 | 3 (2.6%) |
|  | aac(6')-Ib-cr6 | 2 (1.7%) |
| Sulphonamide | sul 2 | 98 (85.2%) |
|  | sul 1 | 36 (31.3%) |
| Trimethoprim | dfrA14 | 69 (60.0%) |
|  | dfrA27 | 17 (14.8%) |
|  | dfrA12 | 14 (12.2%) |
|  | dfrA30 | 10 (8.7%) |
|  | dfrA1 | 3 (2.6%) |
|  | dfrA16 | 2 (1.7%) |
|  | dfrA5 | 1 (0.9%) |
| Tetracycline | tet(A) | 64 (55.6%) |
|  | tet(B) | 2 (1.7%) |
|  | tet(D) | 2 (1.7%) |
