# Supplementary material for: High prevalence of fecal carriage of extended-spectrum beta-lactamase producing Enterobacterales among patients with urinary tract infections in rural Tanzania
Source: Front Microbiol. 2025 Jan 6;15:1517182. doi: 10.3389/fmicb.2024.1517182 (PMC11743186; doi:10.3389/fmicb.2024.1517182)
Supplement: Supplementary file 6 [file Table_5.DOCX]

**Table S5** Plasmid replicons of 115 ESBL *K. pneumoniae* and its related species

| **Classification** | **Replicon type** | **Frequency (n, %). N = 115** |
| --- | --- | --- |
| IncF plasmids | IncFIB(K) | 95 (82.6%) |
|  | IncFII(K) | 39 (33.9%) |
|  | IncFIA(HI1) | 18 (15.7%) |
|  | IncFIB(Mar) | 13 (11.3%) |
|  | IncFIA | 8 (6.9%) |
|  | IncFIB(pKPHS1) | 5 (4.3%) |
|  | IncFIB(pQil) | 3 (2.6%) |
|  | IncFIA(pBK30683) | 1 (0.9%) |
|  | IncFII(pCTU2) | 1 (0.9%) |
|  | IncFII(Yp) | 1 (0.9%) |
| IncH plasmids | IncHI1B | 16 (13.9%) |
| IncR plasmids | IncR | 42 (36.5%) |
| IncX plasmids | IncX3 | 2 (1.7%) |
| IncY plasmids | IncY | 44 (38.3%) |
| Col plasmids | Col440I | 14 (12.2%) |
|  | Col440II | 4 (3.5%) |
|  | ColRNAI | 5 (4.3%) |
|  | Col(pHAD28) | 1 (0.9%) |
