# Supplementary material for: High prevalence of fecal carriage of extended-spectrum beta-lactamase producing Enterobacterales among patients with urinary tract infections in rural Tanzania
Source: Front Microbiol. 2025 Jan 6;15:1517182. doi: 10.3389/fmicb.2024.1517182 (PMC11743186; doi:10.3389/fmicb.2024.1517182)
Supplement: Supplementary file 7 [file Table_6.DOCX]

**Table S6** ESBL E. cloacae molecular patterns

| **Isolate**  **ID** | **ST** | **Plasmid replicons** | **Beta-lactamases** | **Quinolones** | **Aminoglycosides** | **Sulfonamide** | **Tetracycline** | **Trimethoprim** |
| --- | --- | --- | --- | --- | --- | --- | --- | --- |
| DAMRST 0190 | Unknown | IncFIB(K), IncFIB(Mar),  IncHI1B,IncR | SHV-48, OXA-1,  **CTX-M-15** | qnrB1 | aac(3)-IIe, aac(6')-Ib-cr6 | sul1, sul2 |  | dfrA14, dfrA15 |
| DAMRST 220 | Unknown | IncHI2,  IncHI2A | TEM-1, OXA-1,  **CTX-M-15** | qnrB1 | aac(3)-IIe, aac(6')-Ib-cr6, aph(3'')-Ib, aph(6)-Id | sul2 | tet(A) | dfrA14 |
| DAMRST 0274 | 922 | IncFIA | **CTX-M-15** | qnrS1 |  |  |  |  |
| DAMRST 0407 | 1 | IncFIA |  | qnrB4 | aph(6)-Id, aph(3'')-Ib | sul1 |  |  |
| DAMRST 0499 | 171 | IncFIB(pECLA),  IncFII(pECLA) | TEM-1, OXA-1,  **CTX-M-15** | qnrB1 | aadA1, aph(6)-Id, aph(3'')-Ib, aac(3)-IIa, aac(6')-Ib-cr6 | sul2 | tet(A) | dfrA14 |

**Key**

aac (6’) -Ib-cr6 = Fluoroquinolones and aminoglycoside
